# Intra-Articular Injection of Alginate-Microencapsulated Adipose Tissue-Derived Mesenchymal Stem Cells for the Treatment of Osteoarthritis in Rabbits

**DOI:** 10.1155/2018/2791632

**Published:** 2018-06-26

**Authors:** Seongjae Choi, Jun-Hyung Kim, Jeongho Ha, Bo-Ing Jeong, Yun Chan Jung, Geun-Shik Lee, Heung-Myong Woo, Byung-Jae Kang

**Affiliations:** ^1^College of Veterinary Medicine and Institute of Veterinary Science, Kangwon National University, Chuncheon 24341, Republic of Korea; ^2^KPC, Gwangju 12773, Republic of Korea

## Abstract

We investigated the effects of intra-articular injections of alginate-microencapsulated adipose tissue-derived mesenchymal stem cells (ASCs) during osteoarthritis (OA) development in a rabbit model of anterior cruciate ligament transection (ACLT). We induced OA in mature New Zealand white rabbits by bilateral ACLT. Stifle joints were categorised into four groups according to intra-articular injection materials. Alginate microbeads and microencapsulated ASCs were prepared using the vibrational nozzle technology. Two weeks after ACLT, the rabbits received three consecutive weekly intra-articular injections of 0.9% NaCl, alginate microbeads, ASCs, or microencapsulated ASCs, into each joint. Nine weeks after ACLT, we euthanised the rabbits and collected bilateral femoral condyles for macroscopic, histological, and immunohistochemical analyses. Macroscopic evaluation using the modified OA Research Society International (OARSI) score and total cartilage damage score showed that cartilage degradation on the femoral condyle was relatively low in the microencapsulated-ASC group. Histological analysis of the lateral femoral condyles indicated that microencapsulated ASCs had significant chondroprotective effects. Immunohistochemically, the expression of MMP-13 after the articular cartilage damage was relatively low in the microencapsulated-ASC-treated stifle joints. During the development of experimental OA, as compared to ASCs alone, intra-articular injection of microencapsulated ASCs significantly decreased the progression and extent of OA.

## 1. Introduction

Degenerative joint disease or osteoarthritis (OA) of the knee is the most common form of arthritis and reduces quality of life by causing pain, stiffness, and physical disability [1]. OA is characterised by progressive deterioration of cartilage and destruction of the extracellular matrix owing to an impaired anabolic and/or catabolic balance [2, 3]. Articular cartilage has lower capacity for self-repair [4]. It is challenging to enhance regeneration of hyaline cartilage tissue [5]. Many clinical treatments have been used for OA management [6]. These treatments include nonsteroidal anti-inflammatory drugs, platelet-rich plasma, analgesics, hyaluronic acid, mevastatin, and mesenchymal stem cells (MSCs) [7–11]. Although many treatments with MSCs have been developed, adipose tissue-derived mesenchymal stem cells (ASCs) have several advantages. In humans, intra-articular injection of ASCs improves the functioning and reduces pain and cartilage defects of the knee joint [11]. In addition, ASCs can be more easily cultured and obtained in vastly greater quantities—by less aggressive methods—than MSCs can [12–14].

Systemic or local stem cell-based therapies represent a growing field of treatment of OA, resulting in repair of articular cartilage [15]. Nonetheless, studies have increasingly revealed poor viability and a low survival rate of the transplanted stem cells at the disease-affected site [16, 17]. Recent research has therefore focused on enhancing cell viability at a local site affected by the disease and on treatments involving the paracrine effect of MSCs [18–21]. Most therapeutic effects of MSCs are thought to act in a paracrine manner by promoting angiogenesis, tissue regeneration, and production of soluble anti-inflammatory factors [16, 22, 23]. Alginate-microencapsulated cells provide a mechanical barrier that acts as an artificial extracellular matrix, increasing cell viability and allowing for a release of stem cell-produced growth factors and anti-inflammatory factors into surrounding injured tissue [24].

We investigated the effects of periodic intra-articular injection of alginate-microencapsulated ASCs on OA in a rabbit model of anterior cruciate ligament transection (ACLT). We hypothesised that the microencapsulated ASCs would reduce OA progression more effectively than ASCs alone would.

## 2. Materials and Methods

### 2.1. Animals

Fifteen adult New Zealand white rabbits (weighing 3.0–3.5 kg, 25–30 weeks old, male) with closed epiphyses were used in this study. Eleven rabbits were allocated to the OA model and four rabbits to allogeneic-ASC harvesting. The animal care and research protocol was reviewed and approved by the Institutional Animal Care and Use Committee of Kangwon National University (KW-170315-1).

### 2.2. The Rabbit Model of ACLT

Eleven rabbits were anesthetised via intramuscular injection of tiletamine-zolazepam (20 mg/kg; Zoletil 50, Virbac, Seoul, Korea) and xylazine hydrochloride (5 mg/kg; Rompun, Bayer Korea, Seoul, Korea). Intravenous ketoprofen (3 mg/kg; SCD Pharm, Seoul, Korea) was given for preemptive analgesia. Intravenous cefazolin (22 mg/kg; Chong Kun Dang Pharmaceutical, Seoul, Korea) was administered (to prevent infection) at the time of the surgical procedure and once every 24 h for 3 d postoperatively. When the rabbits were under anesthesia, electric clippers were employed to shear the hair overlying each stifle joint, and a sterile surgical operation was carried out to obtain subcutaneous adipose tissue. After the patellar tendon was dislocated laterally, the anterior cruciate ligament of bilateral hind limbs was transected completely with a No. 11 scalpel blade. After suturing, 10 cranial draw motions provoked joint stimulation, and ACLT was completely confirmed. The rabbits were closely monitored for infection and other complications and were allowed to rest in a cage without immobilisation.

### 2.3. ASC Isolation and Culture

Rabbit ASCs were isolated by the collagenase perfusion technique according to previously described methods [22, 25, 26]. Approximately 5 g of fat collected from the posterior cervical subcutaneous adipose tissue of four rabbits was mixed and washed with phosphate-buffered saline (PBS). The adipose tissue was cut into strips during 10 min incubation in collagenase I (Thermo Fisher Scientific, Waltham, MA, USA) in a 6-well plate. Collagenase was dissolved in PBS so that its concentration would be 0.1% in 25 mL and was used to digest adipose tissue at 37°C for 90 min in a water bath. The mixture was shaken every 10 min during the digestion period. Immediately after the reaction was finished, 25 mL of high-glucose Dulbecco's modified Eagle's medium (DMEM, Invitrogen, Carlsbad, CA, USA) was added to neutralise the collagenase activity. The resulting solution was filtered through a 100 *μ*m cell strainer. The filtrate was centrifuged at 1500 ×g for 6 min at 25°C, and the supernatant was removed. Next, a pellet of ASCs was seeded in cell culture dishes and cultured under standard conditions in high-glucose DMEM supplemented with 10% of foetal bovine serum (FBS, Invitrogen, Carlsbad, CA, USA) and 1% of an antibiotic-antimycotic solution (Thermo Fisher Scientific, Waltham, MA, USA). The medium was replaced at 48 h intervals until the cells became confluent. After the cells reached 90% confluence, they were harvested with 0.25% trypsin ethylenediaminetetraacetic acid (EDTA; Welgene, Gyeongsan-si, Korea) and stored in liquid nitrogen or subcultured. When cell density was ~2.5 × 10^4^ cells/cm^2^, passaging of ASCs was performed. The cryopreserved ASCs were used up to passage 5.

### 2.4. Preparation of Alginate Microbeads

A 1.2% sodium alginate solution served as the polymer. A sterile filtered isotonic 1.8% sodium alginate solution (Büchi Labortechnik AG, Flawil, Switzerland) was diluted to 1.2% concentration with sterile normal saline. The 1.2% sodium alginate solution was prepared in syringes. Alginate microbeads were produced by the vibrational nozzle technology by means of a Büchi Encapsulator B-395 Pro (Büchi Labortechnik AG, Flawil, Switzerland) at the parameters previously reported for preparation of alginate microbeads suitable for passing through a 23-gauge needle: nozzle size, 120 *μ*m; frequency, 1000 Hz; flow rate, 23 mL/min; and electrode potential, 1000 V [21]. The gelling solution was prepared in 100 mM calcium chloride. Alginate droplets were gelled in 100 mM calcium chloride for 30 min. The finished alginate microbeads were filtered through a 40 *μ*m cell strainer and washed in PBS. The morphology and size of the alginate microbeads were analysed under a light microscope.

### 2.5. Preparation of Alginate-Microencapsulated ASCs

An alginate solution containing ASCs was prepared by mixing the 1.2% sodium alginate solution with ASCs at a concentration of 10^7^ cells/mL. This suspension was subjected to microencapsulation and was then filtered under optimised processing parameters. Next, the microencapsulated ASCs were washed in 1x PBS, and the size and shape of the microcapsules containing ASCs were analysed by bright-field microscopy.

### 2.6. The Study Scheme

**Figure 1:**
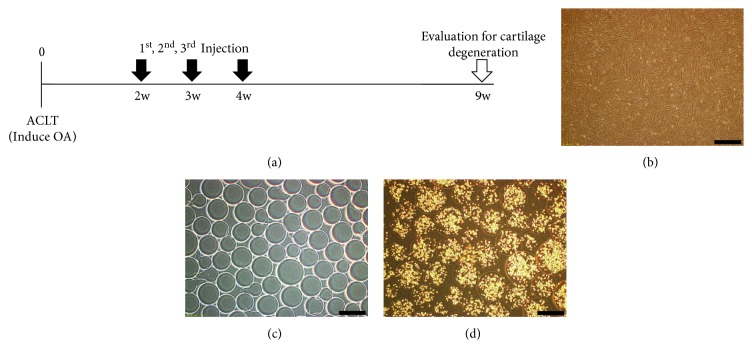
The scheme of the study on intra-articular injection with a material according to experimental groups and microscopic appearance. (a) The study scheme for intra-articular injection of a material according to an experimental group. (b) A light-microscopy image of ASCs having spindle-shaped morphology. (c) A light-microscopy image of alginate microbeads in the absence of cells. (d) Light-microscopic appearance of microencapsulated ASCs at the time of implantation, with 300–400 cells within each 400–500 *μ*m capsule. Scale bar = 500 *μ*m.

Rabbits were injected every week for 3 weeks with a material according to an experimental group, starting at 2 weeks after the ACLT. At 9 weeks after ACLT, all the rabbits were euthanised, and stifle joint samples were collected and evaluated (Figure 1(a)).

### 2.7. Intra-Articular Injections of Materials

Before intra-articular injection of any material, each rabbit was anesthetised with an intramuscular injection of 10 mg/kg tiletamine-zolazepam (Zoletil 50, Virbac, Seoul, Korea) and 5 mg/kg xylazine hydrochloride (Rompun, Bayer Korea, Seoul, Korea). A 1 mL plastic syringe was loaded with one of the materials in 0.5 mL of 0.9% NaCl, with or without the cell suspension; to this syringe, a 21-gauge hypodermic needle was then attached.

For matched-pair analysis, 11 rabbits (22 knees) were used. At 2, 3, and 4 weeks after ACLT, in five rabbits, ASCs were injected into the left stifle joint, and microencapsulated ASCs were injected into the right stifle joint, via the medial approach. In six rabbits, 0.9% NaCl was injected into the left stifle joint, and alginate microbeads were injected into the right stifle joint, via the medial approach. Each rabbit was held down for 5 min to allow for attachment of the injected materials to the synovium.

### 2.8. Macroscopic Analysis

Eleven rabbits were euthanised at 9 weeks after ACLT, by intravenous injection of a potassium chloride overdose. Both femoral condyles were carefully collected to avoid damage to any cartilage surfaces. The articular surfaces of the joints were stained with India ink (American MasterTech, Lodi, CA, USA) for macroscopic examination and were studied regarding fibrillation and erosion [27]. The cartilage surface was painted with India ink twice, and we rinsed the cartilage with PBS each time; the ink was applied 3 min after the rinsing. Macroscopic pictures were taken using a Sony digital camera (Sony, Tokyo, Japan).

The macroscopic OA score was calculated by summing the medial and lateral condyle scores and by means of the modified version of the Osteoarthritis Research Society International (OARSI) scoring system (Supplementary Table 1) [28].

To determine the total cartilage damage score (TCDS), femoral condyles were graded based on ink retention; quantification of a defect region in femoral condyles was conducted in ImageJ (NIH, Bethesda, MD, USA): image analysis software. The TCDS was calculated by adding cartilage damage scores (CDSs) of the medial and lateral femoral condyles. These CDSs were calculated by multiplying the percentage of the damaged articular cartilage area of each condyle by an ink retention-based grade (grade 1, intact surface: surface normal in appearance and not retaining India ink; grade 2, fibrillation: surface retains India ink as elongated specks or light grey or black patches; and grade 3, erosion: loss of exposed cartilage). CDSs ranged from 0 to 300 (0 = no cartilage damage; 300 = complete exposure of subchondral bone). TCDSs were computed via the following equations [29]:

(1) $$\begin{array}{c} CDS=\left[{\%}_{area}\times ink\;retention\;{grade}_{area}\right],\\ TCDS={CDS}_{medial\;femoral\;condyle}+{CDS}_{lateral\;femoral\;condoyle}. \end{array}$$

The macroscopic OA score and TCDS were determined by two veterinarians, one of whom was blinded to the injection material.

### 2.9. Histological Analysis

For the histological examination, both distal femoral cartilages were fixed in a 10% neutral buffered formalin solution (Thermo Fisher Scientific, Waltham, MA, USA) after macroscopic analysis. The specimens were decalcified in a 4% EDTA solution and embedded in paraffin. Paraffin-embedded sections were cut to a thickness of 4 *μ*m in the parasagittal plane. The sections were made through the most severely degenerated area. All the samples were mounted onto slides and stained with haematoxylin and eosin for general pathological observations. In addition, all the samples were stained with Safranin-O and counterstained with fast green so that OARSI scores could be determined. One histopathological section per condyle was independently evaluated under a light microscope (Olympus Optical Co., Tokyo, Japan) by two veterinarians who were blinded to the group distribution. Articular cartilage changes were evaluated according to the OARSI guidelines for evaluation of rabbit tissues [28, 30]. The OA score was an index of the combined grade and stage as seen in a microscopic section (score = grade × stage). The grade represented one of six levels: surface intact, discontinuity, vertical fissures, erosion, denudation, or deformation. The stage was assigned on the basis of the horizontal extent of the involved cartilage surface defect of the underlying OA grade. Stage 1 represents less than 10% involvement, stage 2 means 10–25% involvement, stage 3 denotes 25–50% involvement, and stage 4 represents more than 50% involvement.

### 2.10. Immunohistochemical (IHC) Analysis

From each block, serial sections were cut to 4 *μ*m thickness and mounted onto gelatin-coated slides for improved attachment. The slides were deparaffinised in xylene and next washed in ethanol (100%, followed by 95%) and PBS. Articular cartilage IHC analysis was performed manually by means of a mouse anti-human MMP-13 monoclonal antibody (1 : 20; Thermo Fisher Scientific, Waltham, MA, USA). For antigen retrieval, the sections were incubated and then processed in an autoclave for 5 min at 95°C using 0.1% sodium citrate buffer (pH 6.0). After that, the slides were rinsed three times with PBS and were incubated with the peroxidase-conjugated polymer of the DAKO Real Envision/HRP rabbit/mouse/kit (REAL EnVision Detection System K5007, DAKO, Copenhagen, Denmark) for 30 min, again rinsed three times with PBS, and incubated with DABb from the kit for 5 min, according to the manufacturer's instructions. Finally, nuclei were counterstained with haematoxylin for 30 s, blued under tap water, washed in ethanol, and cover-slipped. A semiquantitative analysis was performed on the cartilage of lateral femoral condyles. Immunoreactive cells and normal cells were counted in the articular surface regions as cells per square millimetre. IHC values, as a percentage of cells positive for MMP-13, were determined for a complete assessment of protein expression, with a maximum score of 100%. The assay was conducted by two blinded investigators [31].

### 2.11. Statistical Analysis

This analysis was performed in Prism 5.00 (GraphPad Software Inc., San Diego, CA, USA). The results are presented as mean ± standard deviation (SD). Statistical significance was assessed by the Kruskal-Wallis test followed by the Mann–Whitney post hoc test (to compare the groups). Data with *P* < 0.05 were considered significant for all tests.

## 3. Results

### 3.1. Morphological Confirmation of ASCs, Alginate Microbeads, and Alginate-Microencapsulated ASCs

Using a light microscope, we confirmed that ASCs were spindle-shaped (Figure 1(b)). Alginate microbeads and microcapsules containing ASCs were also confirmed by light microscopy to have a mostly normal spherical shape. We determined differences in bead diameter between alginate microbeads and microcapsules containing ASCs. The alginate microbeads were 250–300 *μ*m in diameter, and the microcapsules containing ASCs were 400–500 *μ*m in diameter. Each microcapsule containing ASCs included 300–400 cells, and the number of contained cells varied according to the bead diameter (Figures 1(c) and 1(d)).

### 3.2. Macroscopic Analysis

**Figure 2:**
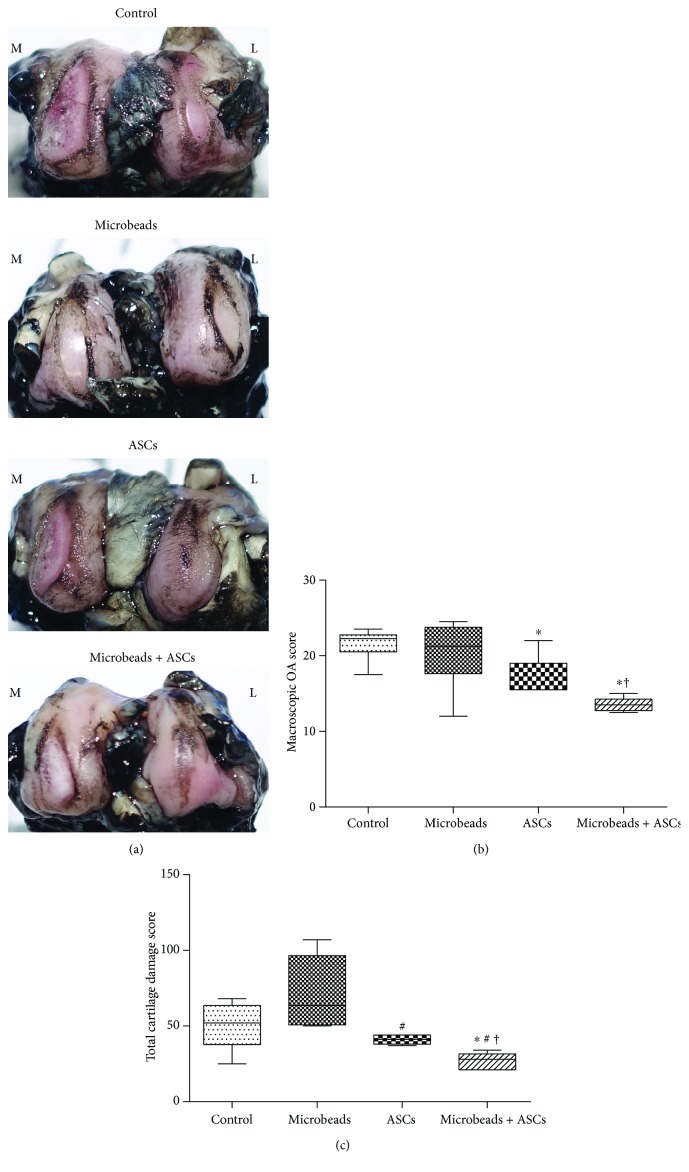
Macroscopic analysis of femoral condyles at 9 weeks after ACLT. (a) A representative specimen of a condyle (from each group) stained with India ink to identify any fibrillation and erosion. (b) The macroscopic OA score. (c) The TCDS. ^∗^A significant difference from the control group (*P* < 0.05). ^#^A significant difference from the alginate microbead group (*P* < 0.05). ^†^A significant difference from the ASC group (*P* < 0.05).

After gross visual inspection, all stifle joints showed complete transection of the anterior cruciate ligament and severe erosion or fissure in both medial and lateral femoral condyles. There were more extensive lesions in medial condyles than in lateral condyles. The grade of the bilateral condyles was “erosion” in the control and alginate microbead groups. Meanwhile, the grade of medial condyles was erosion, and the grade of lateral condyles was “fissure” in the ASC group and in the microencapsulated-ASC group. In addition, the areas of damaged cartilage in the microencapsulated-ASC group appeared to be significantly smaller than those in the other groups.  Figure 2(a) presents examples of a condyle typical of each group, stained with India ink. Significant decreases in the macroscopic OA score were observed in the microencapsulated-ASC group (13.5 ± 0.94) and the ASC group (16.9 ± 2.86) compared with the control group (21.58 ± 2.11). Besides, the macroscopic OA score in the microencapsulated-ASC group was significantly lower than that in the ASC group. Nonetheless, there were no significant differences between the control and alginate microbead groups (20.33 ± 4.48) (Figure 2(b)). Significant decreases in the TCDS were observed in the microencapsulated-ASC group (26.6 ± 5.59) relative to the control group (50.17 ± 15.89), alginate microbead group (71.33 ± 23.99), and ASC group (41 ± 3.08) (Figure 2(c)). The lower macroscopic OA score and TCDS in the microencapsulated-ASC group indicated significantly less damage to the cartilage surface.

### 3.3. Histological Analysis

**Figure 3:**
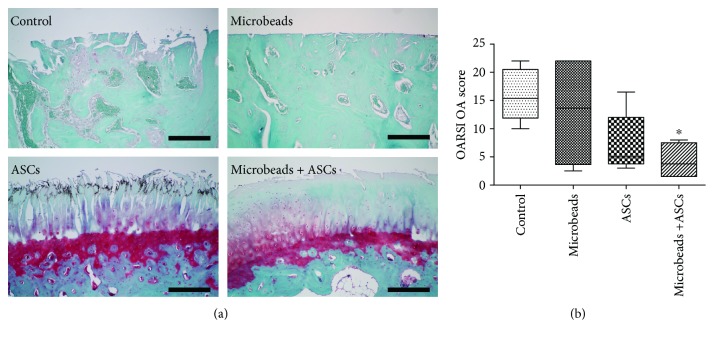
Histological analysis of femoral lateral condyles. (a) A representative specimen of a lateral condyle from each group stained with Safranin-O and counterstained with fast green. (b) The OARSI score of OA. ^∗^A significant difference from the control group (*P* < 0.05). Scale bar = 200 *μ*m.

This evaluation of stifle joints showed more specific differences in lateral condyles (Figure 3(a)). Medial condyle cartilage surfaces showed mostly denudation or deformation, and there were no significant differences among the groups. In lateral condyles, there was no significant difference in the OARSI OA score between the control group (15.88 ± 4.50) and the other groups except for the microencapsulated-ASC group (4.35 ± 3.04) (Figure 3(b)). In the control group and alginate microbead group (12.96 ± 9.14), the lateral articular surface showed denudation or deformation. Although not reaching statistical significance, more favourable scores for the articular surface were observed in the microencapsulated-ASC group than in the ASC group (7.3 ± 5.34).

### 3.4. IHC Analysis

**Figure 4:**
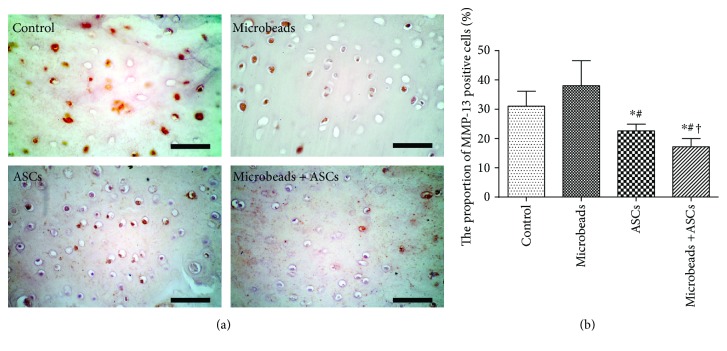
IHC analysis for MMP-13 in cartilage. (a) A representative specimen from each group evaluated for MMP-13 in a femoral lateral condyle. (b) The proportion of MMP-13-positive cells. ^∗^A significant difference from the control group (*P* < 0.05). ^#^A significant difference from the alginate microbead group (*P* < 0.05). ^†^A significant difference from the ASC group (*P* < 0.05). Scale bar = 100 *μ*m.

The expression of MMP-13 was semiquantitatively analysed by IHC staining, using samples prepared at 9 weeks after ACLT. IHC analysis of stifle joints revealed more specific differences in the lateral condyle (Figure 4(a)).

The proportion of MMP-13-positive cells was significantly lower in the microencapsulated-ASC group (17.2 ± 2.77) than in the other groups, including the ASC group (22.6 ± 2.30). There was no significant difference between the control group (31 ± 5.14) and the alginate microbead group (38.17 ± 8.73) (Figure 4(b)).

## 4. Discussion

Many strategies for preventing cartilage degeneration and OA have been developed and clinically tested. The nonimmunogenic effectiveness and potent immunosuppressive activity of MSCs have been reported, and allogeneic MSCs can be used safely in OA without immunosuppressive medication [32, 33]. To enhance their effect, to improve MSC viability, and to prolong the survival of MSCs in a harsh microenvironment, MSCs can be combined with traditional treatments, such as injection of hyaluronic acid [34, 35]. Recently, stem cells were reported to disappear by day 7 after intra-articular injection; periodic injection was found to be more effective. Nevertheless, this method requires many cells and repeated injections [36–38]. If the cell viability were increased, it would be possible to further increase the therapeutic effect, while reducing the frequency and size of MSC injections, and this approach may be more cost-effective. When scaffolds and stem cells are applied to bone defects, the therapeutic effect can be increased by augmenting the differentiation potential of MSCs [39]. On the other hand, such scaffolds have not been employed within joints and have been applied invasively. Recently, it was reported that microencapsulation of cells in alginate is a noninvasive and novel method of increasing the viability of cells for joint injection [21]. To increase cell viability, it is most important to protect the injected cells from the host's immune response. One method of protection is to encapsulate cells within a semipermeable system [40] such as alginate. Alginate is a natural biopolymer extracted from brown algae and is known to be biocompatible, relatively nontoxic, and inexpensive [24, 41, 42].

ASCs were recently reported to exert paracrine action, which indirectly stimulates the secretion of bioactive factors such as cytokines and growth factors [31, 43]. The three-dimensional gel-sphere scaffold of alginate microbeads is a porous structure that allows for diffusion of oxygen and nutrients and for the transport of metabolites [24]. These structural features of alginate microbeads may facilitate the paracrine activity of ASCs used in stem cell therapy.

In this study, we performed three periodic injections, since 2 weeks after ACLT, for the treatment of OA. It has been demonstrated that degenerative changes can be observed in early OA at 4 weeks after ACLT [44]. In acute OA, we thought that the stem cells injected at 2, 3, and 4 weeks after the ACLT would not have a direct therapeutic effect (e.g., cartilage regeneration) because the joint damage would not have advanced sufficiently. Nevertheless, if progressive damage of articular cartilage occurred since the 4 weeks after ACLT, the ASCs would be effective in suppressing OA progression via paracrine action rather than via articular cartilage regeneration. In other studies, cells injected into joints have been found to survive for ~7 d [36–38]. Although the present study did not experimentally determine how long microencapsulated ASCs survive in joints, it has been reported elsewhere that subcutaneously injected microencapsulated cells show more viability *in vivo* than do cells directly injected without microencapsulation [18, 21]. On the basis of previous studies, we expected that alginate microencapsulation might prolong the survival of stem cells in joints and improve OA treatment efficacy by prolonging the paracrine activity.

Macroscopic analysis was performed at 9 weeks after ACLT because all signs of OA were present during the 8–12 weeks after ACLT [44]. When the tibial plateau was observed, OA lesions in this area were mild or moderate. This is a phenomenon that takes place in the ACLT model in rabbits, in agreement with a study by Mero et al. [45]. Given that the lesion is mild or moderate, the tibial plateau appears to be an unreasonable indicator for assessing cartilage degeneration. In addition, there were no differences among the groups in the gross evaluation. Furthermore, acute lesions with ACLT have the greatest effect on the femoral condyle, and some studies also indicate that the femoral condyle is a good indicator of cartilage degeneration [44]. For this reason, we focused on lesions on the femoral condyle.

Stifle joints are often used in OA models. Load distribution and gait mechanics of stifle joints vary depending on animal species. Unlike other animals, rabbits tend to be loaded on the lateral aspect of the stifle joint [46]. Mild early arthritic changes begin to appear from the 4th week after ACLT, and severe cartilage degeneration first occurs in the lateral femoral condyle, followed by the medial femoral condyle and meniscus [45]. Therefore, the lateral compartment in the stifle joint of a rabbit is the site for confirming early cartilage changes [47]. In the lateral femoral condyle, where early cartilage degeneration took place, cartilage damage was significantly lower in the microencapsulated-ASC group than in the other groups. Nonetheless, we grossly evaluated the meniscus, and the damage had already occurred. In addition, when the meniscus was assessed by the grading method of Adams et al. [48], there was no significant difference among the experimental groups. This finding suggests that early administration of microencapsulated ASCs may have prevented or delayed cartilage damage on the lateral femoral condyle, and this damage represents early joint lesions. In contrast, microencapsulated ASCs did not prevent or slow down the damage to the medial femoral condyle and meniscus damage that later developed during OA progression. It is expected that microencapsulated ASCs will survive longer than ASCs alone will, but early repeated administration will not prevent cartilage damage for long periods after administration. Therefore, additional administration after 4 weeks or shortening the harvest time may prevent the damage to the meniscus and to the medial femoral condyle.

India ink staining was performed, and there was an advantage that could be confirmed more visually. There was no significant difference between the control group and the alginate microbead group, implying that alginate microbeads did not afford additional antiarthritis effects. We interpret the differing results between the microencapsulated-ASC group and the ASC group as indicative of a delay in arthritis progression. Various studies suggest that alginate microencapsulation can increase the viability of stem cells and prolong their secretion of therapeutic cytokines [49]. Accordingly, macroscopic evaluation showed that cartilage degeneration significantly decreased in the microencapsulated-ASC group. By contrast, histological evaluation revealed no significant difference in the medial-condyle defects among all the groups, because of the extent of damage. In addition, the microencapsulated-ASC group showed a significant difference in lateral joints only in comparison with the control group. The alginate microbead group manifested large variation in results. Nonetheless, because alginate microbeads are biocompatible, these results do not appear to be due to the characteristics of alginate microbeads. The variation in the results can be assumed to be caused by insufficient sample size and limitations of the model of experimentally induced OA. If the harvest time had been slightly earlier, we may have observed great differences in the OA progression. Further studies on injection timing and frequency in acute and chronic OA should indicate how microencapsulated ASCs can be applied more efficaciously.

The degree of damage to hyaline cartilage can be assessed by IHC staining regarding upregulation of MMP-13, a proven matrix-degrading enzyme in articular cartilage [50]. As previously reported, the hyaline cartilage matrix is composed of type II collagen and the proteoglycan macromolecule aggrecan. When type II collagen is damaged by OA, matrix-degrading enzymes including MMP-1, MMP-8, and MMP-13 are upregulated [51, 52].

In this study, to confirm the effect of microencapsulated ASCs, we identified only MMP-13 expression via IHC analysis. The MMP-13-positive cell ratio was significantly lower in the microencapsulated-ASC group when compared to all the other groups. These results suggest that microencapsulation may increase the paracrine action exerted by ASCs. Assays of additional enzymes and factors, such as collagenase-generated cleavage neoepitope of type II collagen, which has been identified as a sensitive OA biomarker [53, 54], may confirm the influence of microencapsulation on ASCs.

In humans, the effect of arthritis treatment has been found to depend on the dose of stem cells injected. A high-dose group shows clinical, radiological, and arthroscopic results that are more favourable than those from groups receiving a low or medium dose [11]. These data indicate that a sufficient number of MSCs should be delivered to the disease-affected site for the best results. The importance of the cell dose has been raised by several authors [55–57]. Some have reported that injection of 10^7^ MSCs leads to complete healing of scars in rats [55], whereas others have demonstrated that insufficient numbers of applied MSCs yield inferior results [58]. It is expected that alginate microencapsulation will increase the viability of stem cells in joints. Over the course of repeated injections, the number of surviving cells in the microencapsulated-ASC group should be higher than that in the ASC group. This viability may presumably mean that microencapsulated ASCs are more effective than ASCs in the treatment of arthritis.

The limitations of this study are as follows: it is necessary to study the long-term cartilage protection effect because it reflects the short-term cartilage protection activity in the early stages of OA. There is also a need to study potential complications over long periods. In addition, viability of alginate-microencapsulated cells has already been investigated, and in vivo studies have shown that injected alginate-microencapsulated cells are superior to free cells in terms of survival and retention [21]. Nevertheless, because the present study did not investigate the degradation kinetics of alginate microbeads within joints, it is necessary to study microcapsule degradation duration in joints to evaluate the effect in a joint before clinical application. Finally, many types of codelivery strategies are currently being developed and discussed [59, 60]. Therefore, it is necessary to demonstrate that when compared with other codelivery strategies, alginate microbeads ensure higher viability and retention of cells.

## 5. Conclusions

Microencapsulated ASCs slowed the progression of OA and decreased its extent, more so than did free ASCs. Alginate-based microencapsulation may increase ASC viability within the knee joint. To our knowledge, this study is the first to employ alginate-based scaffolding for improving the efficacy of ASC-based treatment of arthritis.

## Supplementary Materials

Supplementary Materials The Modified Osteoarthritis Research Society International (OARSI) scoring system for assessment of macroscopic changes in articular cartilage using India ink.
