# Supplementary material for: Intra-Articular Injection of Alginate-Microencapsulated Adipose Tissue-Derived Mesenchymal Stem Cells for the Treatment of Osteoarthritis in Rabbits
Source: Stem Cells Int. 2018 Jun 26;2018:2791632. doi: 10.1155/2018/2791632 (PMC6038583; doi:10.1155/2018/2791632)
Supplement: Supplementary Materials — The Modified Osteoarthritis Research Society International (OARSI) scoring system for assessment of macroscopic changes in articular cartilage using India ink. [file 2791632.f1.docx]

**Supplementary Table 1.**

The Modified Osteoarthritis Research Society International (OARSI) scoring system for assessment of macroscopic changes in articular cartilage using India ink.

| Articular cartilage macroscopic score | Structure |
| --- | --- |
| 1 | Intact surface^a^ |
| 2 | 0 mm < Fissure ≤ 2 mm |
| 3 | 2 mm < Fissure ≤ 4 mm |
| 4 | 4 mm < Fissure ≤ 6 mm |
| 5 | 6 mm < Fissure ≤ 8 mm |
| 6 | 8 mm < Fissure ≤ 10 mm |
| 7 | 10 mm < Fissure^b^ |
| 8 | 0 mm < Erosion ≤ 2 mm |
| 9 | 2 mm < Erosion ≤ 4 mm |
| 10 | 4 mm < Erosion ≤ 6 mm |
| 11 | 6 mm < Erosion ≤ 8 mm |
| 12 | 8 mm < Erosion ≤ 10 mm |
| 13 | 10 mm < Erosion^c^ |

The measurements represent the length of a lesion.

^a^Intact surface: surface normal in appearance and not retaining India ink.

^b^Fissure: surface retains India ink as elongated specks or light grey patches.

^c^Erosion: loss of cartilage exposing the underlying bone.
